# A Weighted Genetic Risk Score Predicts Surgical Recurrence Independent of High-Risk Clinical Features in Dupuytren’s Disease

**DOI:** 10.1097/PRS.0000000000005208

**Published:** 2019-01-29

**Authors:** Sophie A. Riesmeijer, Oliver W. G. Manley, Michael Ng, Ilja M. Nolte, Dieuwke C. Broekstra, Paul M. N. Werker, Dominic Furniss

**Affiliations:** Groningen, The Netherlands; and Oxford, United Kingdom; 1 From the Departments of Plastic Surgery and Epidemiology, University of Groningen, University Medical Center Groningen; and the Botnar Research Centre, Nuffield Department of Orthopaedics, Rheumatology and Musculoskeletal Sciences, Oxford University.

## Abstract

**Background:**

Dupuytren’s disease is a very common, highly heritable palmar fibromatosis. In a recent genome-wide association study, 26 single-nucleotide polymorphisms were found to be associated with development of Dupuytren’s disease. The authors generated a weighted genetic risk score based on the genotype at these single-nucleotide polymorphisms. In two independent cohorts, they tested the association among high weighted genetic risk score, clinical features that predict a high risk of recurrence, and recurrence after surgery.

**Methods:**

Clinical data were obtained from patient questionnaires and clinical records, with missing data accounted for by imputation. Genotyping was performed as part of the recent genome-wide association study. Logistic regression was performed to study the association among weighted genetic risk score, high-risk clinical features, and recurrence, with a weighted genetic risk score analyzed as a continuous variable, and also grouped into four categories.

**Results:**

Using univariable logistic regression, a high weighted genetic risk score was associated with the presence of all high-risk clinical features: early age of onset, bilateral disease, ectopic disease, and a positive family history (*p* ≤ 0.004). After multivariable logistic regression accounting for these factors, an increased weighted genetic risk score was still associated with the need for repeated Dupuytren’s disease surgery (*p* = 0.004).

**Conclusions:**

The authors’ results suggest that a weighted genetic risk score is useful in predicting the risk of disease recurrence, and may be used by surgeons to personalize prognostication. In the future, a weighted genetic risk score may be useful for determining the most appropriate initial surgical procedure in patients with Dupuytren’s disease.

**CLINICAL QUESTION/LEVEL OF EVIDENCE:**

Risk, III.

## Abstract 2

Supplemental Digital Content is available in the text.

**Figure:**
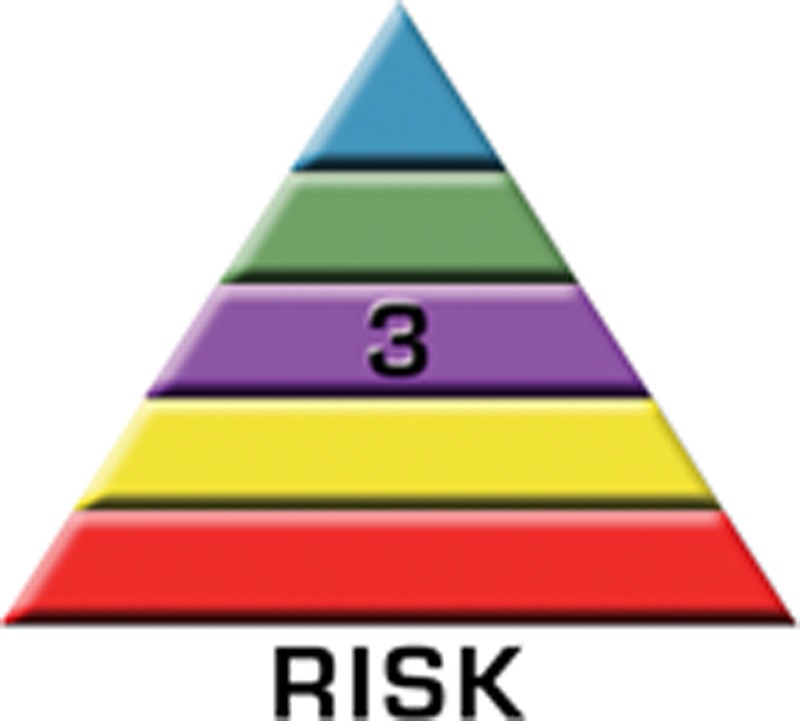


Dupuytren’s disease is a fibroproliferative disorder of the palmar fascia. Diseased palmar fascia forms nodules and cords that extend into the digits. The diseased tissue can eventually contract, causing limited mobility and loss of function.^1^ Standard treatment is aimed at correcting flexion deformities through surgical incision or excision, or collagenase treatment.^2^ Despite many patients having good outcomes from treatment, Dupuytren’s disease remains incurable, and surgical recurrence rates following treatment are high, varying from 21 to 85 percent at 5 years.^3^ Surgical complication rates in revision surgery are high.^4^ There are several known clinical features that predict a more aggressive disease course and higher risk of recurrence in Dupuytren’s disease: age of onset before age 50 years, bilateral disease, ectopic disease outside of the hand, and positive family history of Dupuytren’s disease. Together, these factors are termed the Dupuytren’s diathesis.^5^

Dupuytren’s disease is known to be a complex disease with several genetic and environmental risk factors, each contributing to susceptibility to Dupuytren’s disease. Environmental factors associated with Dupuytren’s disease include smoking, alcohol intake, diabetes, hyperlipidemia, and epilepsy.^6^ Twin and sibling studies demonstrate the genetic basis of Dupuytren’s disease, and heritability has been estimated at 80 percent.^7,8^ A pilot genomewide association study identified nine single-nucleotide polymorphisms associated with Dupuytren’s disease. Several of these variants were found on loci containing genes involved in the Wnt-signaling pathway, suggesting that abnormalities in this pathway may predispose to Dupuytren’s disease.^9^ A recent genomewide association study has demonstrated an additional 17 single-nucleotide polymorphisms associated with a susceptibility to Dupuytren’s disease, further confirming the genetic basis of this disease.^10^

Genetic risk scores are useful tools that can be generated following a genome-wide association study to summarize the risks from multiple variants into a single variable. The basic premise is that a person with many risk-increasing alleles will have greater susceptibility to a disease, and thus a higher genetic risk score, than someone with only a few risk-increasing alleles. Genetic risk scores have been created in a number of diseases with complex genetic origins such as diabetes, where a weighted genetic risk score was able to differentiate between type 1 and type 2 diabetes mellitus in adults, and monogenic and type 1 diabetes mellitus in neonates.^11,12^ The effects of each predisposing allele are assumed to be additive, and each effect allele is weighted by the natural logarithm of the odds ratio for that allele.

Following the pilot genome-wide association study, a weighted genetic risk score comprising only nine variants was shown to be associated with Dupuytren’s diathesis factors in a Dutch cohort.^13^ We hypothesized that a new genetic risk score, constructed using the extended results from the recent genomewide association study, would be associated with diathesis in a different cohort. Furthermore, we hypothesized that a weighted genetic risk score would also predict a higher risk of disease recurrence after surgery, thus representing a novel prognostic indicator that might be used in presurgical counseling. We report here on results in two independently ascertained cohorts from the United Kingdom and The Netherlands, showing an association between a weighted genetic risk score, Dupuytren’s diathesis, and recurrence after surgery.

## PATIENTS AND METHODS

### Ethical Approval

Each study was approved by the research ethics committee or equivalent at all institutions where the work was carried out: the British Society for Surgery of the Hand Genetics of Dupuytren’s Disease study (United Kingdom; Oxfordshire Research Ethics Committee B/09/H0605/65), and the Genetic Origin of Dupuytren Disease and Associated Fibromatosis study (The Netherlands; Medical Ethics Committee 2007/067). All participants provided written informed consent.

### Patient Populations

U.K. patients were recruited as part of the British Society for Surgery of the Hand Genetics of Dupuytren’s Disease study, and controls were recruited from the U.K. Household Longitudinal Study, as described previously.^9^ Patients in this study were recruited from U.K. hand surgery centers, and must have had at least one operation for Dupuytren’s disease. Patients in the Dutch Genetic Origin of Dupuytren Disease and Associated Fibromatosis study were also recruited from hand surgery clinics; this recruitment has also been described previously.^9^

### Clinical Data

Patients included in the British Society for Surgery of the Hand Genetics of Dupuytren’s Disease study completed two questionnaires. An initial questionnaire assessed the presence of high-risk features that are part of the Dupuytren’s diathesis, namely, presence of ectopic fibrotic lesions (Ledderhose or Peyronie disease), bilateral disease, age of onset younger than 50 years, and positive family history. A second questionnaire recorded details of the number of operations patients had undergone on each hand. Recurrence in the U.K. study was defined as having had more than one operation on the same hand.

Data on diathesis features from Dutch participants were collected by a surgeon at entry into the study. Clinical data on recurrence were extracted from medical notes. Recurrence in the Dutch study was defined as a patient having undergone more than one operation on the same finger of the same hand, or having a clinical diagnosis of recurrence from the treating surgeon.

### Genetic Risk Score

DNA was obtained from patients’ saliva (British Society for Surgery of the Hand Genetics of Dupuytren’s Disease study) and blood (Genetic Origin of Dupuytren Disease and Associated Fibromatosis study), and genotyping was performed as described.^10^ A weighted genetic risk score was calculated using the following formula:


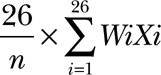


where *i* is the single-nucleotide polymorphism, *n* is the number of single-nucleotide polymorphisms genotyped for that patient, *W_i_* is the weight for single-nucleotide polymorphism *i* (the natural log of the odds ratio for Dupuytren’s disease), and *X_i_* is the number of risk alleles the patient carries of that single-nucleotide polymorphism.^14^ To allow for comparison between patients when some single-nucleotide polymorphisms were missing, the weighted genetic risk score was divided by the total number of single-nucleotide polymorphisms genotyped and multiplied by the total of 26 single-nucleotide polymorphisms.

### Imputation and Statistical Analysis

Statistical analysis was performed in Stata MP14 (StataCorp, College Station, Texas) and R (R Foundation for Statistical Computing, Vienna, Austria). Quantile-quantile plot was used to demonstrate the normal distribution of the weighted genetic risk score data. The Levene test was performed to assess the equality of variances. The two-tailed *t* test was performed to test for differences between the means of weighted genetic risk score between cases and controls, and between male and female patients.

Before imputation, in the British Society for Surgery of the Hand Genetics of Dupuytren’s Disease study, we excluded 48 samples that had been genotyped at a single single-nucleotide polymorphism, and would therefore violate the missing at random assumption. We then accounted for missing clinical data and weighted genetic risk score by imputation in our remaining 6126 individuals using multiple imputation by chained equations.^15^ We imputed missing data on family history, early onset, bilateral disease, recurrence, sex, and weighted genetic risk score. Ectopic disease was not imputed, as there were no missing data. We created 75 imputed data sets, each using 100 cycles of imputation, with the following input variables: recurrence, weighted genetic risk score, family history, early onset, ectopic disease, bilateral disease, and sex. In the Genetic Origin of Dupuytren Disease and Associated Fibromatosis study, we imputed the missing data using the same method as in the British Society for Surgery of the Hand Genetics of Dupuytren’s Disease study, with the additional variable ectopic disease included in the imputation chain.

After imputation, we analyzed 6126 individuals in the British Society for Surgery of the Hand Genetics of Dupuytren’s Disease study and 1730 individuals in the Genetic Origin of Dupuytren Disease and Associated Fibromatosis study. Univariable logistic regression was performed to test for associations between each diathesis feature and recurrence in the British Society for Surgery of the Hand Genetics of Dupuytren’s Disease study. We analyzed weighted genetic risk score in two ways: first, in four groups with mean − 1 SD, mean, and mean + 1 SD as thresholds; second, as a continuous variable. We used multivariable logistic regression to test for associations between category of weighted genetic risk score, diatheses, and recurrence. We used univariable logistic regression to test for the association between increasing value of weighted genetic risk score and recurrence. Meta-analysis was performed using the fixed effect inverse variance method. A value of *p* < 0.05 was considered statistically significant.

## RESULTS

### Dupuytren’s Diathesis

High-risk features of the Dupuytren’s diathesis were common in both cohorts (Table 1). The ratio of male to female patients was approximately 3:1, and the percentage of individuals with each feature was broadly similar across cohorts. Bilateral disease was the most common diathesis feature, reported at 63 percent and 40 percent in the British Society for Surgery of the Hand Genetics of Dupuytren’s Disease study and the Genetic Origin of Dupuytren Disease and Associated Fibromatosis study, respectively. The least common diathesis feature was ectopic disease, reported at less than 13 percent in both cohorts.

**Table 1.**
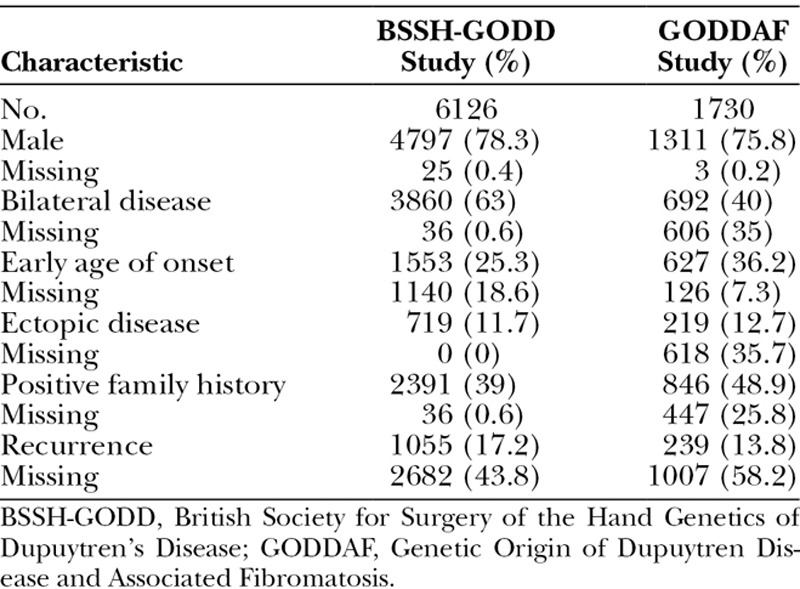
Clinical Features of the Two Cohorts

### Weighted Genetic Risk Score

After genomewide association study quality control, the majority of individuals from the discovery cohort of the British Society for Surgery of the Hand Genetics of Dupuytren’s Disease study were successfully genotyped at all 26 single-nucleotide polymorphisms. In the Genetic Origin of Dupuytren Disease and Associated Fibromatosis cohort, the majority were directly genotyped at 22 loci. (**See Table, Supplemental Digital Content 1**, which shows the distribution of weighted genetic risk score single-nucleotide polymorphism count in the British Society for Surgery of the Hand Genetics of Dupuytren’s Disease study and the Genetic Origin of Dupuytren Disease and Associated Fibromatosis study, http://links.lww.com/PRS/D260.)

In the British Society for Surgery of the Hand Genetics of Dupuytren’s Disease study, the calculated weighted genetic risk score ranged from 3.48 to 9.76, with a mean of 6.32. As expected, cases had a higher mean weighted genetic risk score than controls (6.32 versus 5.44; *p* < 0.001). Within cases, women had a higher mean weighted genetic risk score than men (6.42 versus 6.29; *p* < 0.001). In the Genetic Origin of Dupuytren Disease and Associated Fibromatosis study, weighted genetic risk score ranged from 3.00 to 9.77, with a mean of 6.26. Here, women had a mean weighted genetic risk score similar to that for men (6.27 versus 6.25; *p* = 0.70). In both cohorts, weighted genetic risk score was distributed normally. [**See Figure, Supplemental Digital Content 2**, which shows the quantile-quantile plot, demonstrating the Gaussian nature of the weighted genetic risk score distribution in both (*above*) the British Society for Surgery of the Hand Genetics of Dupuytren’s Disease study and (*below*) the Genetic Origin of Dupuytren Disease and Associated Fibromatosis study. *wGRS*, wighted genetic risk score, http://links.lww.com/PRS/D261.]

### Weighted Genetic Risk Score Is Associated with Dupuytren’s Diathesis in the British Society for Surgery of the Hand Genetics of Dupuytren’s Disease Study

In the British Society for Surgery of the Hand Genetics of Dupuytren’s Disease cohort, multinomial logistic regression analysis demonstrated significant associations between each diathesis factor and the two highest weighted genetic risk score categories (Table 2). The odds ratios of expressing a particular Dupuytren’s diathesis feature consistently increased for each additional unit of weighted genetic risk score (Table 3).

**Table 2.**
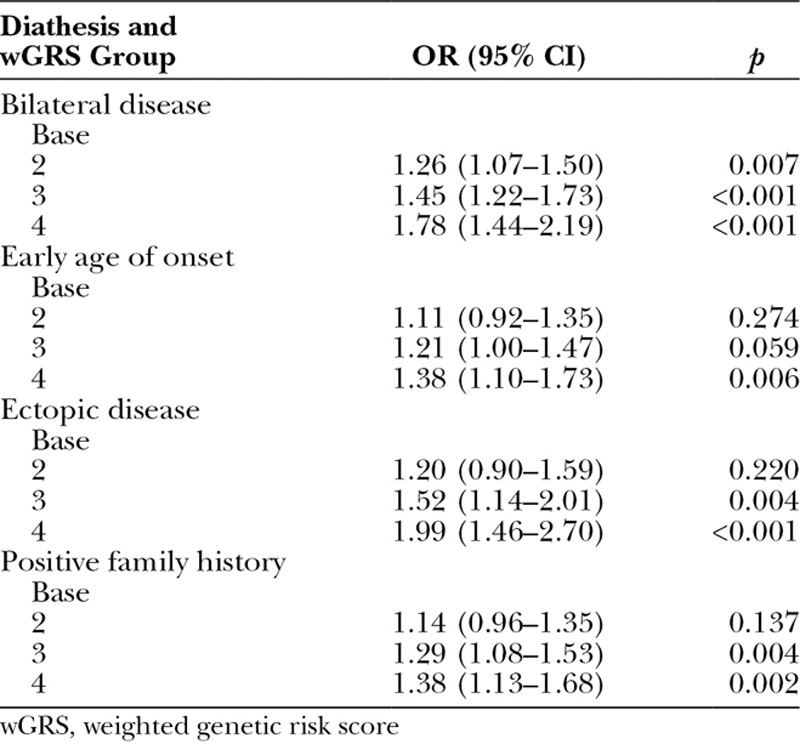
Association between Dupuytren’s Diathesis and Weighted Genetic Risk Score in the British Society for Surgery of the Hand Genetics of Dupuytren’s Disease Cohort

**Table 3.**
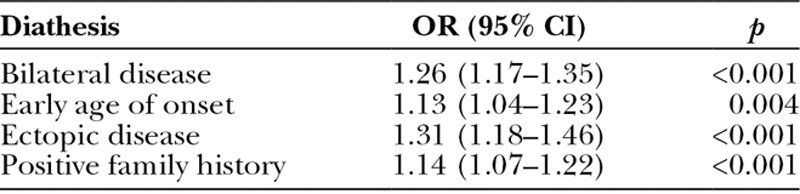
Odds of Expressing a Dupuytren’s Diathesis Feature for Each Increasing Unit of Weighted Genetic Risk Score in the British Society for Surgery of the Hand Genetics of Dupuytren’s Disease Cohort

### Dupuytren’s Diathesis Is Associated with Recurrence in the British Society for Surgery of the Hand Genetics of Dupuytren’s Disease Cohort

Each diathesis feature predicted higher odds of requiring further surgery in the British Society for Surgery of the Hand Genetics of Dupuytren’s Disease study: early age of onset was the strongest predictor (OR, 2.38; 95 percent CI, 2.00 to 2.83; *p* < 0.001) (Table 4). The odds of further surgery also increased with increasing number of diathesis features (Table 5).

**Table 4.**
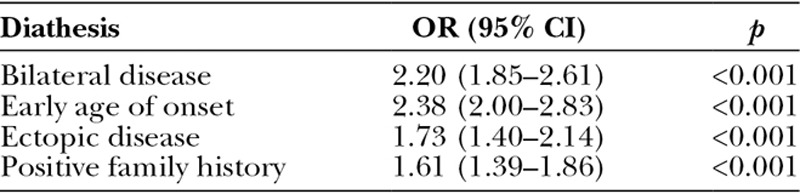
Odds Ratio of Recurrence for Each Diathesis Feature in the British Society for Surgery of the Hand Genetics of Dupuytren’s Disease Cohort

**Table 5.**
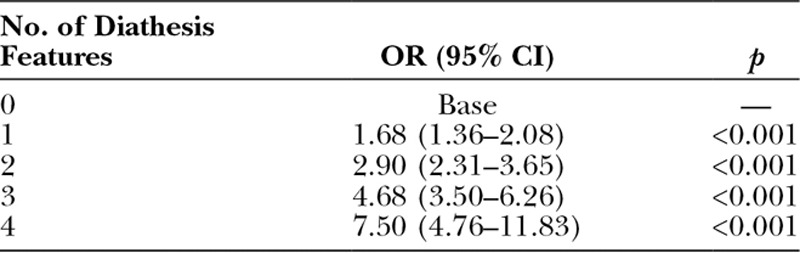
Odds Ratio of Recurrence for Increasing Number of Diathesis Features in the British Society for Surgery of the Hand Genetics of Dupuytren’s Disease Cohort

### Weighted Genetic Risk Score Is Associated with Recurrence in the British Society for Surgery of the Hand Genetics of Dupuytren’s Disease and the Genetic Origin of Dupuytren Disease and Associated Fibromatosis Cohorts

In the British Society for Surgery of the Hand Genetics of Dupuytren’s Disease cohort, both in unadjusted models and after adjustment for bilateral disease, ectopic disease, early onset, and positive family history, recurrence increased with increasing weighted genetic risk score category (Table 6). In the highest weighted genetic risk score category, the adjusted odds ratio for recurrence was 1.46 (95 percent CI, 1.09 to 1.96; *p* = 0.008). With each unit increase in weighted genetic risk score, the odds ratio for recurrence was 1.18 (95 percent CI, 1.06 to 1.31; *p* = 0.002) (Table 7).

**Table 6.**
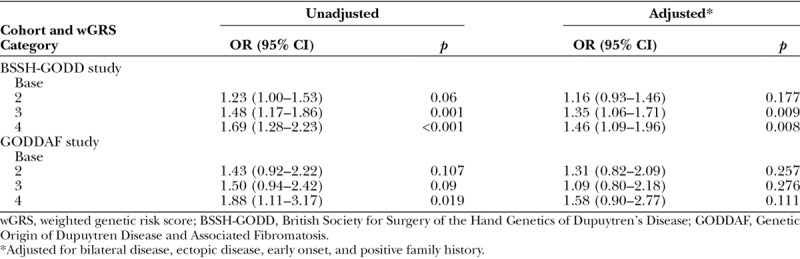
Risk of Dupuytren’s Disease Recurrence by Weighted Genetic Risk Score Category in the British Society for Surgery of the Hand Genetics of Dupuytren’s Disease and Genetic Origin of Dupuytren Disease and Associated Fibromatosis Cohorts

**Table 7.**
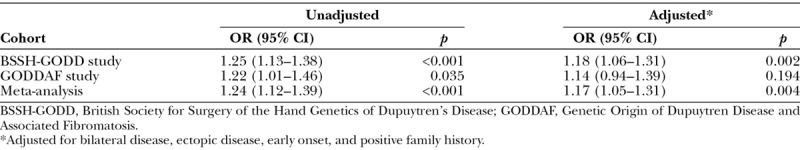
Increased Risk of Recurrence for Each Unit Increase in Weighted Genetic Risk Score

In the Genetic Origin of Dupuytren Disease and Associated Fibromatosis cohort, recurrence was also correlated with weighted genetic risk score in unadjusted models, with an odds ratio of 1.88 (95 percent CI, 1.11 to 3.17; *p* = 0.019) between the highest and lowest categories. With each unit increase in weighted genetic risk score, the increased odds ratio for recurrence was 1.22 (95 percent CI, 1.01 to 1.46; *p* = 0.035) (Table 6). However, when we adjusted for diathesis factors, these results lost statistical significance, most likely because of the small size of this cohort. Despite this, the magnitude and direction of the association with recurrence is consistent across cohorts (Table 7).

## DISCUSSION

In this study, we show that patients with high weighted genetic risk scores are more susceptible to recurrence following surgery for Dupuytren’s disease. Importantly, this was demonstrated in patients from two different populations, ascertained through independently designed studies. Using logistic regression, we demonstrated that the increased risk of recurrence predicted by weighted genetic risk score is independent of clinical features known to associate with aggressive, highly recurrent disease—the Dupuytren’s diathesis.

Previous work based on the results of our pilot genome-wide association study had demonstrated an association between an early version of the weighted genetic risk score, composed of nine single-nucleotide polymorphisms, and some features of Dupuytren’s diathesis (i.e., ectopic disease, early age of onset, and positive family history).^13^ This study confirms and extends this association in a larger cohort, to include bilateral disease.

Genetic risk scores work on the assumption that each included single-nucleotide polymorphism is independently associated with an increased risk for the tested variable. Using a weighted genetic risk score, rather than testing each single-nucleotide polymorphism individually, has more power because the effects of the single-nucleotide polymorphisms are summed, and no multiple testing correction is needed. Our genetic risk score was a weighted model rather than an additive model, in which the presence of each single-nucleotide polymorphism would equally increase the score. This weight was determined on the basis of the odds ratio of the single-nucleotide polymorphism when considering predisposition to Dupuytren’s disease as a whole. This makes the assumption that a single-nucleotide polymorphism contributes to diathesis and recurrence with a similar weight to its overall predisposition to disease. Therefore, one weakness of our weighted genetic risk score is that these single-nucleotide polymorphisms were selected because they are known risk factors for Dupuytren’s disease—they are not necessarily risk factors for recurrence or diathesis per se. Including single-nucleotide polymorphisms that may not associate with recurrence increases the noise and consequently reduces the power of our model.

In our study, we used two definitions of recurrence as made necessary by the different methods of data collection for the two cohorts. No universal definition of recurrent Dupuytren’s disease exists.^16,17^ The definition of recurrence in the Genetic Origin of Dupuytren Disease and Associated Fibromatosis study, being multiple operations in the same finger of the same hand, will not include patients who rejected further treatment after surgery, or patients who had further treatment in a different hospital that was not detected by our study. The definition in the British Society for Surgery of the Hand Genetics of Dupuytren’s Disease study is a second operation on the same hand. This may exclude individuals who refuse further surgery for recurrent disease, and it will also include instances where patients have undergone surgery in stages, with different fingers operated on at different times. Although our definitions have limitations, it is compelling that two separate definitions of recurrence led to consistent results. Our conclusions remain important for patient counseling. If a patient has a high weighted genetic risk score, and/or many diathesis features are present, they are more likely to have an aggressive disease course and require repeated surgery.

A further limitation of our work is that we are unable to provide detailed analysis of subgroups of patients (e.g., classified by initial severity of contracture or initial surgical treatment). Furthermore, we cannot correlate our weighted genetic risk score to the time from initial treatment to recurrence. These outstanding questions are interesting areas for future research.

With improved access to whole-genome sequencing in clinical settings, counseling on the basis of genetic risk scores may soon become realistic for a variety of surgical diseases. Presently, patients with Dupuytren’s disease are counseled based on clinical diathesis factors only. Including weighted genetic risk score may improve the accuracy of counseling, especially as many patients present to clinicians before the full extent of their diathesis has become apparent. More work is needed to confirm how patients with different weighted genetic risk scores respond to different treatment modalities. This will be another step along the road to personalized treatment for our patients.

## CONCLUSIONS

We have demonstrated that a weighted genetic risk score predicts recurrence after surgery in Dupuytren’s disease, a very common complex fibroproliferative disease of the palmar fascia. This predictive value is independent of known high-risk clinical features. Knowledge of this weighted genetic risk score might facilitate more accurate prognostication for the individual patient, and in the future may influence the choice of initial treatment.

## ACKNOWLEDGMENTS

Dominic Furniss, D.M., is supported by an Intermediate Clinical Fellowship from the Wellcome Trust (097152/Z/11/Z). This work was supported by the National Institute for Health Research Biomedical Research Centre, Oxford. The funders had no role in study design, data collection and analysis, decision to publish, or preparation of the manuscript. The authors are grateful to all affected and control individuals who participated in the study. The authors wish to thank the collaborators of both the British Society for Surgery of the Hand Genetics of Dupuytren’s Disease group and the Genetic Origin of Dupuytren Disease and Associated Fibromatosis group for recruitment of cases. The data sets generated and analyzed during the current study are available in the European Genome-phenome Archive repository (EGAS00001001206, phs000303.v1.p1, and EGAS00000000043).

## Supplementary Material
