# Supplementary material for: A Weighted Genetic Risk Score Predicts Surgical Recurrence Independent of High-Risk Clinical Features in Dupuytren’s Disease
Source: Plast Reconstr Surg. 2019 Jan 29;143(2):512–8. doi: 10.1097/PRS.0000000000005208 (PMC6358194; doi:10.1097/PRS.0000000000005208)
Supplement: Supplementary file 1 [file prs-143-0512-s001.pdf]

Supplementary Table 1. Distribution of wGRS SNP count in BSSH and GODDAF.

	**Frequency**	
**SNP Count**	**BSSH-GODD**	**GODDAF**
19	5	1
20	3	9
21	8	114
22	61	1336
23	1060	-
24	2	-
25	58	-
26	3808	-
Missing	1121	270
**Total**	**6126**	**1730**
