# Supplementary material for: A Weighted Genetic Risk Score Predicts Surgical Recurrence Independent of High-Risk Clinical Features in Dupuytren’s Disease
Source: Plast Reconstr Surg. 2019 Jan 29;143(2):512–8. doi: 10.1097/PRS.0000000000005208 (PMC6358194; doi:10.1097/PRS.0000000000005208)

Supplementary Figure 1. Quantile-quantile plot demonstrates the Gaussian nature of the wGRS distribution in both a) GODD and b) GODDAF.

a)

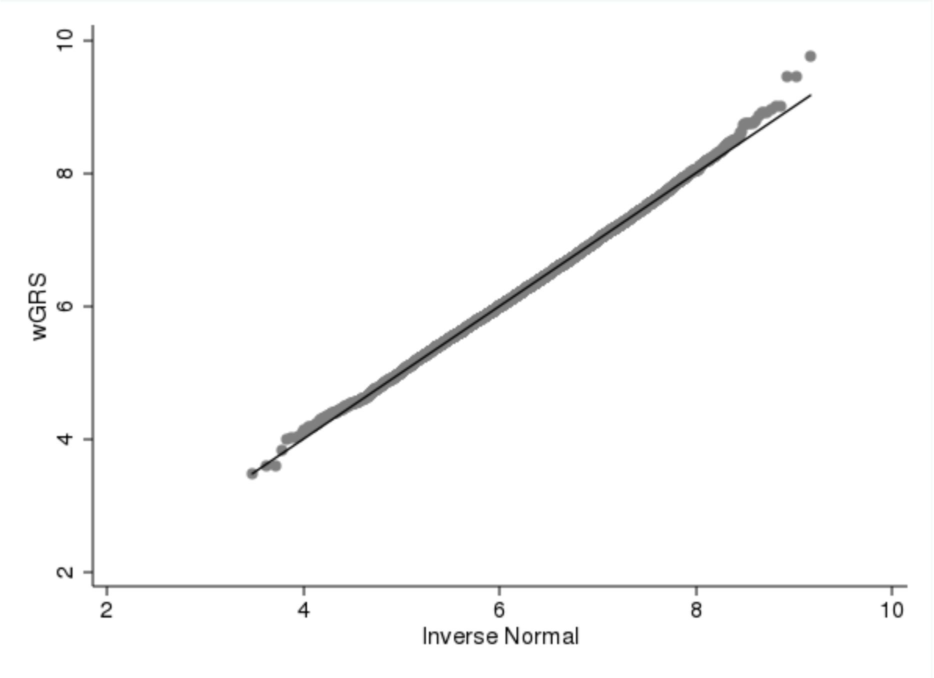

b)

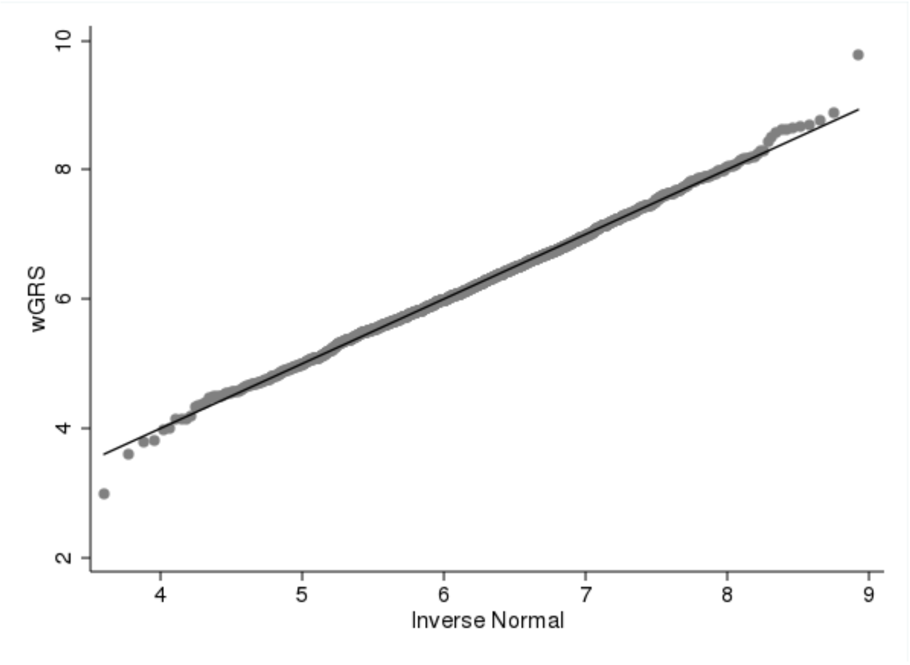

Supplement: Supplementary file 2 [file prs-143-0512-s002.pdf]
